# Incidence and risk factors for acute kidney injury after total joint arthroplasty

**DOI:** 10.1186/s42836-022-00120-z

**Published:** 2022-05-03

**Authors:** Chun Wai Hung, Theodore S. Zhang, Melvyn A. Harrington, Mohamad J. Halawi

**Affiliations:** 1 Department of Orthopedic Surgery, Baylor College of Medicine, Houston, TX USA; 2 Baylor College of Medicine, Houston, TX USA

**Keywords:** Acute kidney injury, Arthroplasty, Bundle buster, Incidence, Optimization

## Abstract

**Background:**

Acute kidney injury (AKI) is one of the most common medical causes for readmission following total joint arthroplasty (TJA). This study aimed to (1) examine whether the incidence of AKI has changed over the past decade with the adoption of modern perioperative care pathways and (2) identify the risk factors and concomitant adverse events (AEs) associated with AKI.

**Methods:**

535,291 primary TJA procedures from the American College of Surgeons National Surgical Quality Improvement Program from 2011 to 2018 were retrospectively reviewed. The annual incidence of AKI was analyzed for significant changes over time. Matched cohort analyses were performed to identify the risk factors and AEs associated with AKI using multivariate logistic regression.

**Results:**

The mean incidence of AKI was 0.051%, which remained unchanged during the study period (*P* = 0.121). Factors associated with AKI were diabetes (OR 1.96, *P* = 0.009), bilateral procedure (OR 6.93, *P* = 0.030), lower preoperative hematocrit level (OR 1.09, *P* = 0.015), body mass index (OR 1.04, *P* = 0.025), and higher preoperative BUN (OR 1.03, *P* = 0.043). AKI was associated with length of stay (LOS) > 2 days (OR 4.73, *P* <  0.001), non-home discharge (OR 0.25, P <  0.001), 30-day readmission (OR 12.29, *P* <  0.001), and mortality (OR 130.7, *P* <  0.001).

**Conclusions:**

The incidence of AKI has not changed over the past decade, and it remains a major bundle buster resulting in greater LOS, non-home discharge, readmissions, and mortality. Avoidance of bilateral TJA in patients with DM and high BMI as well as preoperative optimization of anemia and BUN levels are advised.

## Introduction

To successfully navigate bundled payment programs for total joint arthroplasty (TJA), it is paramount to avoid perioperative adverse events (AEs), commonly termed “bundle busters” [1]. AEs can result in additional treatments that significantly increase the 90-day episode-of-care costs [2]. Aside from prosthetic infections, medical complications are the most common reasons for readmission following TJA, accounting for 41-51% of the economic burden for 90-day readmissions [3–5]. In particular, acute kidney injury (AKI) is the 4th most common and 4th most expensive medical cause for 90-day readmission following TJA [5].

The incidence of AKI after TJA has been reported to range from 0.3 to 16.2% [6–11]. While a number of risk factors for AKI following TJA have been identified, our current state of knowledge is limited by studies largely based on single institutions, small patient samples, and inconsistent diagnosis criteria [6, 8]. It is also unclear to what extent the adoption of modern-day perioperative practices, such as spinal anesthesia and multimodal analgesia, have had an impact on the incidence of AKI [12]. Multimodal analgesia based non-steroidal anti-inflammatory drugs (NSAIDs) has emerged as an important tool for perioperative pain management strategies to reduce opioid reliance [13]. The concerns of AKI in the setting of known renal toxicity from NSAIDs combined with spinal anesthesia-induced hypotension beg the need for more contemporary studies on AKI after TJA [14, 15].

The objectives of this study were (1) to examine the annual incidence of AKI over the past decade, (2) identify the risk factors for development of AKI, and (3) to better characterize the effects of AKI on bundled payment models in terms of associated cost drivers. This knowledge is critical to help orthopedic surgeons devise strategies to guide preoperative risk stratification and optimization, in order to mitigate AKIs and navigate bundled payment programs.

## Methods

This study was exempt from institutional review board (IRB) approval. A retrospective review of the American College of Surgeons National Surgical Quality Improvement Program (ACS-NSQIP) database was performed from 2011 to 2018. The ACS-NSQIP database is a large validated national database including over 700 hospitals frequently used in quality-related joint arthroplasty studies [16–18]. Patients undergoing elective primary total knee and hip arthroplasty (TKA, THA) were identified based on current procedural terminology (CPT) codes 27,447 and 27,130 respectively. Emergent, non-elective, tumor-related, and revision procedures were excluded. Acute kidney injury (AKI) was an independent parameter collected by the ACS-NSQIP database among other postoperative quality metrics.

Demographic information was collected on patients, including age at time of surgery, sex, body mass index (BMI), and race/ethnicity (White, non-Hispanic White, non-Hispanic Black, Hispanic, or Asian). Comorbidities included tobacco use within 1 year prior to surgery, chronic steroid use, diabetes mellitus (DM), hypertension (HTN), chronic obstructive pulmonary disease (COPD), congestive heart failure, bleeding disorders, history of metastatic cancer, anemia, dyspnea, and chronic kidney disease (CKD). Anemia was defined as hematocrit of < 42% and < 37% for males and females respectively; and CKD was defined as preoperative creatinine > 1.5 mg/dL [19, 20]. Perioperative and laboratory data were also collected. Perioperative data included the indication for surgery (primary *vs*. secondary osteoarthritis), laterality (unilateral or bilateral), American Society of Anesthesiologists’ (ASA) physical classification, and operative time. Laboratory data included sodium, blood urea nitrogen (BUN), creatinine, albumin, bilirubin, aspartate aminotransferase (AST), alkaline phosphatase (ALP), white blood cell count (WBC), hematocrit, platelets, partial thromboplastin time (PTT), international normalized ratio (INR), and prothrombin time (PT) levels.

The primary aims were to (1) calculate the annual incidence of AKI from 2011 to 2018, (2) to identify risk factors for AKI development, and (3) to record adverse outcomes associated with AKI. Metrics of adverse outcomes included length of stay (LOS), discharge destination, 30-day readmission, and 30-day mortality. Univariate mixed effect logistic regression was used to analyze significant differences in AKI annual incidence from year to year. Two patient groups were compared in a 1 to 2 ratio based on propensity matching: those experiencing 30-day AKI and those without AKI (control). The criteria for matching were age, sex, ASA score classification, operation year, preoperative creatinine levels, and CPT code. Matching by ASA score controlled for baseline medical status. The operation year controlled for potential variations in perioperative care pathways. Matching by preoperative creatinine levels minimized the confounding effects of baseline kidney function. Finally, matching by CPT code allowed for direct comparison of TKAs to TKAs and THAs to THAs, thus avoiding the confounding effects attributed to the different procedures. Multivariate analysis was used to identify the concomitant adverse outcomes associated with development of AKI.

Continuous variables were reported as mean and standard deviation, and compared using standard student’s *t*-test. Categorical variables were expressed as absolute frequencies and percentages and compared using Pearson’s Chi-squared test. Two-sided analyses were done for *P* values, and statistical significance was assumed at *P* <  0.05. Demographic, comorbidity, laboratory, and perioperative variables that were significant were included in the multivariate logistic regression analyses. Results for such analysis were presented in odds ratios (OR) and 95% confidence intervals (CI). Data were analyzed using a standard statistical software package, Stata® 16.1 (Stata Corp, 204 College Station, TX).

## Results

A total of 535,291 TJA procedures were analyzed. Overall, the incidence of postoperative AKI was 0.051%. The annual incidence of AKI within the first 30 postoperative days from 2011 to 2018 is shown in Table 1. The annual incidence ranged from 0.04 to 0.10%, with no statistically significant changes over time (*P* = 0.121).

**Table 1:** Annual incidence of acute kidney injury (AKI) within 30 days postoperatively between 2011 and 2018

2011	2012	2013	2014	2015	2016	2017	2018	*P* Value
0.08%	0.05%	0.05%	0.04%	0.04%	0.07%	0.04%	0.05%	0.121

The 274 patients with AKI were matched to 546 TJA patients without AKI. The comparison of patient characteristics of the two groups is shown in Table 2. Those with AKI were more likely to be non-Hispanic Blacks (14.2% *vs*. 6.41%, *P* <  0.001). In terms of comorbidities, those with AKI were more likely to have diabetes (40.2% *vs*. 20.7%, *P* <  0.001), hypertension (88% *vs*. 76.6%, *P* <  0.001), COPD (10.6% *vs*. 5.1%, *P* = 0.004), anemia (52.9% *vs*. 38.1%, *P* <  0.001), dyspnea (17.2% *vs*. 10.3%, *P* = 0.005), and chronic kidney disease (30.7% *vs*. 13.9%, *P* <  0.001). Those with AKI were more likely to have undergone TJA due to underlying diagnosis other than primary osteoarthritis (92% *vs*. 96%, *P* = 0.017), more likely to have had bilateral procedure (3.28% *vs*. 0.92%, *P* = 0.014), or have longer operative time (100.8 min *vs*. 91.3 min, *P* = 0.001). In terms of preoperative labs, those with AKI had higher BUN (27.2 *vs*. 21.4, *P* <  0.001), lower albumin (3.9 *vs*. 4.0, *P* = 0.005), and lower hematocrit (38.5% *vs*. 40.7%, *P* <  0.001) levels.

**Table 2:** Patient characteristics comparing those with and without acute kidney injury

	No Acute Kidney Injury	Acute Kidney Injury	***P*** Value
Demographic Characteristics			
Age (years)	72.5 ± 10.0	71.1 ± 10.2	0.070
Sex (male:female)	317 (58.1%): 229 (41.9%)	146 (53.3%): 128 (46.7%)	0.193
Body Mass Index	31.8 ± 6.7	34.4 ± 7.4	< 0.001
Race/Ethnicity			
Non-Hispanic White	412 (75.5%)	171 (62.4%)	< 0.001
Non-Hispanic Black	35 (6.41%)	39 (14.2%)	< 0.001
Hispanic	16 (2.9%)	8 (2.9%)	0.993
Asian	8 (1.5%)	3 (1.1%)	0.664
Comorbidities			
Tobacco smoking within 1 year	46 (8.4%)	30 (11.0%)	0.240
Chronic steroid use	22 (4.0%)	10 (3.7%)	0.791
Diabetes	113 (20.7%)	110 (40.2%)	< 0.001
Hypertension	418 (76.6%)	241 (88.0%)	< 0.001
COPD	28 (5.1%)	29 (10.6%)	0.004
Congestive heart failure	6 (1.1%)	8 (2.9%)	0.058
Bleeding disorders	27 (5.0%)	20 (7.3%)	0.171
History of metastatic cancer	2 (0.4%)	1 (0.4%)	0.998
Anemia	208 (38.1%)	145 (52.9%)	< 0.001
Dyspnea	56 (10.3%)	47 (17.2%)	0.005
Chronic kidney disease	76 (13.9%)	84 (30.7%)	< 0.001
Perioperative characteristics			
Primary osteoarthritis	524 (96.0%)	252 (92.0%)	0.017
Total Hip Arthroplasty	198 (36.3%)	95 (34.7%)	0.654
Total Knee Arthroplasty	348 (63.7%)	179 (65.3%)	0.654
Bilateral procedure	5 (0.92%)	9 (3.28%)	0.014
ASA Physical Classification	2.9 ± 0.5	3.0 ± 0.6	0.701
Operative time (minutes)	91.3 ± 34.8	100.8 ± 42.2	0.001
Sodium (mEq/L)	139.7 ± 2.9	139.4 ± 3.1	0.114
BUN (mg/dL)	21.4 ± 10.3	27.2 ± 15.0	< 0.001
Creatinine (mg/dL)	1.3 ± 1.5	1.5 ± 1.0	0.132
Albumin (g/dL)	4.0 ± 0.4	3.9 ± 0.4	0.005
Bilirubin (mg/dL)	0.6 ± 0.5	0.8 ± 1.2	0.205
AST (units/L)	23.6 ± 1.08	23.2 ± 12.2	0.741
ALP (units/L)	84.2 ± 33.4	91.2 ± 38.1	0.058
WBC (× 10^9^ cells/L)	7.5 ± 6.2	7.8 ± 3.3	0.377
Hematocrit (%)	40.7 ± 4.7	38.5 ± 5.0	< 0.001
Platelets (× 10^9^ /L)	227.6 ± 60.3	232.4 ± 72.2	0.314
PTT (sec)	29.8 ± 5.2	29.9 ± 5.5	0.913
INR	1.1 ± 0.2	1.1 ± 0.3	0.538
PT (sec)	12.5 ± 3.3	13.2 ± 4.9	0.568

*ALP* Alkaline phosphatase, *ASA* American Society of Anesthesiologists, *BUN* Blood urea nitrogen, *CHF* Congestive heart failure, *CKD* Chronic kidney disease, *COPD* Chronic obstructive pulmonary disease, *INR* International normalized ratio, *PTT* Partial thromboplastin time, *PT* Prothrombin time, *WBC* White blood count

Multivariate logistic regression results are shown in Table 3. After adjusting for baseline factors, the significant risk factors for AKI were BMI (OR 1.04, 95% CI 1.00–1.07, *P* = 0.025), diabetes (OR 1.96, 95% CI 1.19–3.23, *P* = 0.009), bilateral procedure (OR 6.93, 95% CI 1.23–39.12, *P* = 0.030), higher preoperative BUN (OR 1.03, 95% CI 1.00–1.05, *P* = 0.043), and lower preoperative hematocrit (OR 0.92, 95% CI 0.86–0.98, *P* = 0.015).

**Table 3:** Risk Factors for Acute Kidney Injury

Variables	Odds Ratio (95% Confidence Interval)	***P*** Value
Demographics		
Body Mass Index	1.04 (1.00 – 1.07)	0.025
Non-Hispanic White	0.69 (0.37 – 1.30)	0.257
Non-Hispanic Black	1.46 (0.61 – 3.50)	0.397
Comorbidities		
Diabetes	1.96 (1.19 – 3.23)	0.009
Hypertension	1.09 (0.61 – 1.95)	0.762
COPD	1.39 (0.61 – 3.19)	0.437
Anemia	0.76 (0.40 – 1.42)	0.382
Dyspnea	1.00 (0.52 – 1.90)	0.991
Chronic kidney disease	1.20 (0.61 – 2.34)	0.597
Perioperative characteristics		
Bilateral procedure	6.93 (1.23 – 39.12)	0.030
Primary osteoarthritis	0.78 (0.30 – 2.05)	0.619
Preoperative BUN	1.03 (1.00 – 1.05)	0.043
Preoperative albumin	0.99 (0.56 – 1.72)	0.961
Preoperative hematocrit	0.92 (0.86 – 0.98)	0.015
Dependent functional status	2.73 (0.84 – 8.90)	0.096
Operative time	1.00 (1.00 – 1.01)	0.363

*AKI* Acute kidney injury, *BMI* Body Mass Index, *BUN* Blood urea Nitrogen, *COPD* Chronic obstructive pulmonary disease, *THA* Total Hip Arthroplasty, *TKA* Total Knee Arthroplasty. *P*-values were determined using multivariate logistic regression of acute renal failure within 30 days postoperatively controlling for risk factors which were significant in univariate analyses. Total hip and knee arthroplasty patients without acute kidney injury within 30-days postoperatively were used as controls

As seen in Table 4, the development of AKI was associated with LOS > 2 days (OR 4.73, 95% CI 3.54–6.34, *P* <  0.001), non-home discharge (OR 0.25, 95% CI 0.19–0.34, *P* <  0.001), readmission within 30 days (OR 12.29, 95% CI 7.81–19.35, *P* <  0.001), and mortality within 30 days (OR 130.7, 95% CI 17.96–950.94, *P* <  0.001).

**Table 4:** Additional 30-day adverse events associated with postoperative acute kidney injury

Outcome	Odds Ratio (95% Confidence Interval)	***P*** Value
Length of Stay > 2 days	4.73 (3.54 – 6.34)	< 0.001
Discharge to Home	0.25 (0.19 – 0.34)	< 0.001
Readmission within 30 days	12.29 (7.81 – 19.35)	< 0.001
Mortality within 30 days	130.70 (17.96 – 950.94)	< 0.001

## Discussion

In this study, 0.051% of patients developed AKI following TJA, and the rate remained unchanged from 2011 to 2018. Diabetes, bilateral procedure, BMI, higher preoperative BUN levels, and lower preoperative hematocrit were significant risk factors. AKI was associated with prolonged hospitalization, non-home discharge, readmission, and higher mortality.

The overall incidence of AKI in this study was much lower (0.051%) than reported in previous studies (0.3 –16.2%) [7, 10, 12, 21, 22]. In a retrospective review of 8,127,282 TKA patients using Nationwide Inpatient Sample, Singh *et al*. [11] found the incidence of AKI was 1.3%. However, that study was based on a more dated sample (1998–2014) and relied on ICD-9 codes for diagnoses without clearly distinguishing if AKI was present at the time of surgery. In contrast, the key advantage of the ACS-NSQIP database is that it is based on manual chart abstraction, which is less prone to errors due to billing codes. Another possible explanation for the discrepancy in the rates of AKIs is that different measures were used, such as risk, injury, failure, loss of kidney function, and end-stage kidney disease (RIFLE) and acute kidney injury network (AKIN) classifications [6].

The risk factors for AKI based on the multivariate analyses were higher BMI, diabetes, bilateral procedure, higher preoperative BUN and lower preoperative hematocrit. Multiple studies have shown that increased BMI was an independent risk factor for AKI as well as other complications [23, 24]. In an analysis of 22,808 patients from the Veteran Affairs Surgical Quality Improvement Program database, Ward *et al*. [25] showed, via multivariable regression, that BMI > 40 was an independent risk factor for AKI following THA, with OR = 1.79. Similarly, other studies have shown that diabetes and elevated creatinine were also risk factors for the development of AKI [23, 26]. Creatinine level is often used as screening marker for glomerular filtration rate and, therefore, kidney function. In this study, we found that in addition to creatinine, AKI was correlated with higher BUN and lower hematocrit respectively. Lower preoperative hematocrit has been shown to be a risk factor for the postoperative development of AKI in the revision arthroplasty setting [27]. Simultaneous TKA has also been shown previously to pose higher risk for the development of AKI. In a single center retrospective review, Koh *et al*. compared the risk of AKI for those undergoing staggered (< 7 days between procedures), staged (8 days-1 year between procedures), and simultaneous TKA [28]. The simultaneous TKA group had the highest risk of AKI (OR 7.7, *P* <  0.001).

Few studies have examined the change in the incidence of AKI following TJA. One of the cornerstones of multimodal analgesia is the use of NSAIDs [13]. The main drawback of NSAIDs is their potential adverse event profile on the cardiovascular, gastrointestinal, and renal systems [14]. The renal toxicity of NSAIDs is attributed to the inhibition of cyclooxygenase (COX) 1 and 2, which are responsible for the production of prostaglandins that mediate the vasodilation of the renal tubules [15]. NSAIDs have been found to be associated with AKI [15]. However, few studies examined the association of perioperative use of NSAIDs with AKI following TJA. Gharaibeh *et al*. [21] failed to find an association between perioperative NSAIDs use and postoperative AKI, but they noted that this failure might be attributed to confounders, as those with significant comorbidities, such as CKD and heart failure, had significantly less exposure to NSAIDs.

To our knowledge, only two studies examined the change in AKI rates over time following TJA. In a meta-analysis of THA studies reporting incidence of perioperative AKI, Thongprayoon *et al*. [6] found that between 2012 and 2018, there was a trend of decreasing AKI incidence after THA. In a single institution review, Yayac *et al*. showed fairly constant rates of AKI between 2005 and 2017, ranging from 2.1–5.5 %[12] although no statistical analysis was performed. The current study found that AKI rates varied between 0.04% and 0.1% from 2011 to 2018, but there were no statistically significant differences overall. This suggests that AKI incidence has been fairly stable even with the increasing use of recent perioperative advancements such as hypotensive anesthesia and multimodal analgesia.

“Bundle-busters” are drawing scrutiny as the medical system shift towards value-based care since they can make a TJA episode of care financially non-viable. This study showed that the development of AKI within 30 preoperative days is significantly associated with increased LOS, non-home discharge, readmission, and mortality, which all have serious health as well as financial implications. In a single center retrospective case-controlled review of 1719 primary TJA, Abar *et al*. [21] found that AKI was associated with increased LOS and a mean cost differential of $81,781 in total hospital charges. Similarly, in an analysis of the NIS database of patients undergoing TKA, Singh and Cleveland [11] demonstrated that increased risk of complications was associated with AKI, including implant infection, transfusion, revision surgery, death, and longer LOS which translated to higher mean hospital charges, $71,385 *vs*. US$42,067. Risk-adjustment of bundles have previously been advocated for non-modifiable factors such as those undergoing TJA due to oncologic reasons or conversions as well as other socioeconomic factors [29–31]. Therefore, risk adjustment for factors leading to AKI should be given serious consideration in future bundle payment models.

This study is significant because it lends further credence to the notion that AKI is a potential bundle buster complication. TJA, by virtue of being elective procedures, allows orthopedic surgeons to optimize the medical status of patients preoperatively. Surgeons should be aware of the modifiable risk factors that can help avoid postoperative AKI. In a prospective study, Lands *et al*. [32] achieved a significant reduction in AKI rates, after the implementation of a targeted protocol, from 6.3 to 1.2%. Some of the risk factors found in the current study to be associated with the development of AKI can be carefully optimized prior to surgery, namely, avoidance of bilateral procedures and optimization of hematocrit levels especially in patients with diabetes, obesity or lower kidney function. Furthermore, given that diabetes and CKD are non-modifiable risk factors, this highlights the need for risk-adjusted bundle payment programs.

Despite the strengths of this study, including the use of a large validated national sample of patients undergoing TJA, there were several limitations. AKI was identified based on chart review at each individual site. However, there are many different criteria for defining AKI, so using a single one may underestimate its incidence, and the NSQIP database does not provide a clear definition of AKI [6]. Second, although CKD was determined based on preoperative creatinine data, additional qualifiers on severity of CKD was not assessed since the GFR was not available. Furthermore, the adverse events were only collected for up to 30 days postoperatively, so this might underestimate the true rates as well as additional adverse outcomes associated with AKI.

## Conclusions

The annual incidence of AKI after TJA has been stable over the last decade. It is critical to optimize BMI, preoperative hematocrit, and BUN prior to TJA. Arthroplasty surgeons should avoid bilateral procedures and attempt to correct or optimize low hematocrit levels in those with known risk factors for developing AKI, such as diabetes, lower kidney function, and obesity. Furthermore, given that diabetes and CKD are non-modifiable risk factors, this highlights the need for risk-adjusted bundle payment programs. It is important to recognize the financial risks of AKI as a bundle buster, and its health costs in terms of increased hospitalization time, requirements for discharge to higher care facility, readmission, and even mortality.

## Data Availability

The datasets used and/or analyzed during the current study are available from the corresponding author on reasonable request.

## Abbreviations

AKI: Acute kidney injury
TJA: Total joints arthroplasty
AE: Adverse events
LOS: Length of stay
NSAIDs: Non-steroidal anti-inflammatory drugs
CKD: Chronic kidney disease
COPD: Chronic obstructive pulmonary disease
BMI: Body mass index
BUN: Blood urea nitrogen
GFR: Glomerular filtration rate
DM: Diabetes mellitus
HTN: Hypertension
